# Study of platelet‐rich fibrin combined with rat periodontal ligament stem cells in periodontal tissue regeneration

**DOI:** 10.1111/jcmm.13461

**Published:** 2018-01-24

**Authors:** Xuejing Duan, Zhiyong Lin, Xiujuan Lin, Zhiqiang Wang, Yihua Wu, Mei Ji, Wei Lu, Xiaoyang Wang, Dongsheng Zhang

**Affiliations:** ^1^ School of Stomatology Shandong Provincial Hospital affiliated to Shandong University Jinan Shandong Province China; ^2^ School of Stomatology Qianfoshan Hospital affiliated to Shandong University Jinan Shandong Province China

**Keywords:** periodontal regeneration, platelet‐rich fibrin, periodontal ligament stem cells

## Abstract

The objective of this study was to investigate the advantages and feasibility of periodontal tissue regeneration using platelet‐rich fibrin (PRF) combined with rat periodontal ligament stem cells (PDLSCs) for the first time. We first determined the effect of PRF on rat PDLSCs *in vitro*. We next conducted an *in vivo* study, in which a tissue engineering technique was performed to repair periodontal defects in five groups: a blank group, collagen group (implanted collagen membrane), collagen + cells group (implanted collagen membrane and rat PDLSCs), PRF group (implanted PRF membrane) and PRF + cells group (implanted PRF membrane and rat PDLSCs). PRF greatly enhanced cell proliferation, mRNA and protein expression levels of bone sialoprotein (BSP), osteocalcin (OC), and runt‐related transcription factor 2 (RUNX2) and activity of alkaline phosphatase (ALP) *in vitro*. Transplantation of PRF combined with rat PDLSCs resulted in higher expression of osteopontin (Opn), collagen I (COL1A) and RUNX2 at both 12 and 24 days after surgery. Micro‐computed tomography and histological analysis showed substantially more new bone formation in the PRF + cells group at 24 days after surgery. Based on these results, we discuss the role of PRF in the proliferation and differentiation of rat PDLSCs and suggest that PRF combined with rat PDLSCs provides a valuable tool for periodontal tissue engineering.

## Introduction

Periodontal disease is an infectious and destructive chronic disease that can lead to loss of dental support tissue such as the periodontal ligament, alveolar bone and cementum. Moreover, periodontal disease is now recognized as a risk factor for the development of various chronic systemic diseases (*e.g*. coronary heart disease, diabetes), which seriously threaten human health. In recent years, stem cell methods, tissue engineering and related technologies have been developed, providing the opportunity to explore periodontal regeneration 1.

In 2004, for the first time, Seo *et al*. 2 isolated human PDLSCs and found that these cells showed strong proliferative ability and can form natural periodontal tissues *in vivo*, including the cementum, periodontal ligament and alveolar bone, suggesting their use in periodontal tissue engineering. Upon induction *in vitro*, PDLSCs can differentiate into osteoblasts, adipocytes and chondrocytes, not only showing the typical morphological and structural characteristics of these cells, but also expressing their specific surface markers. PDLSCs provide new opportunities for the study of the mechanisms underlying periodontal regeneration, as well as clinical periodontal regeneration treatments, and have thus been of great interest in the area of periodontal disease. At present, PDLSCs have been used in preclinical research, showing a certain degree of progress 3. PDLSCs are directly derived from periodontal tissues and show strong differentiation and proliferation ability, with the capacity to form three different periodontal supporting tissues in the local environment. A systematic review aiming to evaluate mesenchymal stem cell (MSC) periodontal regenerative potential in animal models showed that PDL‐MSC consistently promoted increased periodontal ligament (PDL) and cementum regeneration 4 **.** Therefore, compared to periodontal tissue‐rebuilding techniques that cannot recapitulate periodontal tissue morphology, the use of PDLSCs as seed cells for periodontal tissue engineering can achieve truer regeneration and reconstruction of periodontal tissue.

The basic requirements for a scaffold material for periodontal tissue engineering include good biocompatibility, biodegradability and adjustable degradability; good osteoinduction and osteoconductivity to facilitate cell adhesion and proliferation; and ease in shaping, disinfecting and storage 5. PRF is a second‐generation platelet‐concentrated product, after platelet‐rich plasma (PRP), which was first reported by Choukroun *et al*. in 2001 6. PRF is a three‐dimensional protein gel obtained by the direct centrifugation of autologous peripheral blood and is thus considered an autologous graft 7. Unlike PRP, PRF does not require the addition of biological agents; accordingly, PRF has no toxicity or immunogenicity, is easy and inexpensive to prepare and carries no risk of cross‐infection or associated ethical issues 8. In addition, as a scaffold material, PRF can release cytokines that promote periodontal tissue regeneration. PRF can therefore not only utilize its mechanical effects to postpone platelets, but can also chemically associate with small molecules, such as various cytokines present in the circulation and glucose glycosaminoglycans, resulting in slow and sustained cytokine release during PRF degradation, 9. Together, this suggests that PRF may be the ideal material for tissue regeneration. Several studies have shown that PRF can significantly promote the repair of soft and hard tissue defects caused by hypoplasia, age‐related atrophy, trauma, cancer and other factors 10, 11. The PRF matrix can also promote endothelial cell proliferation and angiogenesis, promoting healing at wound sites 12. PRF can be used to treat gingival recession and to promote tissue healing, reducing patient discomfort 13, 14. In addition, PRF can promote the healing of soft tissue and repair ear cartilage defects, and thus confers protection and plasticity as a bone graft biofilm. Experimental data have shown that PRF can promote the formation of new bone and improve the efficacy and quality of bone defect repair 15, 16, 17, 18. Therefore, as a bone repair material, PRF can promote osteogenesis and bone healing. PRF induces the expression of phosphorylated extracellular‐related kinase, osteoprotegerin and ALP in periodontal ligament fibroblasts, indicating that it promotes periodontal regeneration 19. Recent clinical trials have shown that the use of PRF alone can reduce the depth of the periodontal pocket, as well as the loss of attachment 20, while the combined application of PRF and ultrasound bone cutting technology could effectively and safely promote the healing of alveolar fissures 21. Recent researches showed that PDLSC sheets combined with PRF which were assessed in a nude mouse tended to develop into PDL‐like tissues after 8 weeks. However, there has been no report on the potential application of PRF combined with stem cells for periodontal tissue engineering 22.

In this study, we aimed to experimentally investigate the feasibility of PRF for periodontal tissue engineering by observing the effects of PRF on rat PDLSCs. We further explored the feasibility and advantages of PRF as a matrix material for promoting periodontal tissue regeneration using PRF and PDLSCs to repair periodontal defects in an animal model.

## Materials and methods

### Culture and identification of rat PDLSCs

Freshly extracted dental molars were immediately immersed in alpha‐minimal essential medium (Gibco, New York, USA) containing double antibiotics (100 μg/ml each streptomycin and penicillin G; Sijiqing Company, Hangzhou, China) and washed ten times with phosphate‐buffered saline (PBS, pH 7.2) under sterile conditions. The periodontal ligament cells were then cultured using a tissue enzymatic digestion method. We used a limited dilution method to sort the rat PDLSCs. The morphologies of PDLSCs were observed by inverted phase‐contrast microscopy and identified by vimentin immunofluorescence and chromosome analysis.

### PRF preparation

A 4‐ml blood sample was obtained from the orbital sinus of each rat. The blood sample was collected in a plain tube, which was immediately centrifuged at 1843 g for 10 min. to prepare the PRF gel. In the middle of the tube, a fibrin clot formed between the supernatant acellular plasma and the lower red corpuscle. The gel was placed in between two layers of sterile gauze, and gentle pressure was applied to obtain the PRF membrane.

### Effect of PRF on rat PDLSCs *in vitro*

The PDLSCs were divided into PRF and non‐PRF groups, and proliferation rates were measured by 3‐(4,5‐dimethylthiazol‐2‐yl)‐2,5‐diphenyltetrazolium bromide (MTT) cell proliferation assay at 24, 48, 72, and 96 hrs.

To determine the effect of PRF on gene expression, total RNA was extracted from the cells of both groups 7 and 14 days after differentiation using the RNeasy kit (Qiagen, Valencia, CA, USA) following the manufacturer's instructions. RNA (1 μg) was reverse‐transcribed using the SuperScript First‐Strand Synthesis System (Invitrogen, Carlsbad, CA, USA) following the manufacturer's recommendations, and the cDNA was used in a reverse transcription polymerase chain reaction (RT‐PCR) with iQ SYBR Green Supermix (Bio‐Rad, Hercules, CA, USA). The results were normalized to GAPDH expression.

The effect of PRF on ALP activity was determined using enzyme dynamics methods. Western blot analysis was performed to observe BSP, OC and RUNX2 protein expression 7 and 14 days after differentiation. Finally, the morphology of the PDLSCs was observed using an electron microscope.

### Surgical procedure

The experimental animals were divided into five groups: blank, collagen (implanted collagen membrane), collagen + cells (implanted collagen membrane and rat PDLSCs), PRF (implanted PRF membrane) and PRF + cells (implanted PRF membrane and rat PDLSCs). Fifty‐eight‐week‐old male nude mice (body weight 25–30 g) were used in this experiment. Under general anaesthesia, a periodontal fenestration defect model was established, as previously described 23, 24, 25. In brief, an incision was made bilaterally at the lower border of the mandible, followed by dissection of the underlying muscle, ensuring attachment of the oral mucosa on the superior wall to the intraoral keratinized gingival margin. The bone overlying the first molar, the buccal mandibula, was removed using a tooth bur at high speed under PBS irrigation 0.5 mm away from the coronal top. The roots were carefully denuded of periodontal ligament (PDL), overlying cementum and superficial dentin under a stereomicroscope. An approximately 4 × 3 × 2 mm^3^ periodontal defect was modified, and the surrounding buccal mandibula was removed.

### RT‐PCR of the newly formed tissue

Groups of 25 mice were killed 12 and 24 days after surgery. Tissues from the edges of the defect sites were cut from six mandibular samples (three animals), immediately frozen in liquid nitrogen and stored at −80°C for RT‐PCR. The tissues were then homogenized in TRIzol solution (Invitrogen), followed by the total RNA isolation procedure recommended by the manufacturer. Freshly isolated RNA was reverse‐transcribed into cDNA, and RT‐PCR was performed as described previously (Tu *et al*., 2008; Xu *et al*., 2009).

### Histomorphometry and micro‐computed tomography (CT) scanning analyses

Tissues from the original defect of each group of mice were fixed in 10% formalin at 4°C for 48 hrs and then scanned by micro‐CT (mCT‐35, Scanco Medical AG, Bassersdorf, Switzerland) with a resolution of 7 μm. The samples were then decalcified in 10% ethylenediaminetetraacetic acid for 2 weeks, embedded in paraffin and cut into serial buccal and lingual sagittal cross sections. The sections were stained with haematoxylin and eosin (H&E) according to standard methods. Newly formed bone in the H&E‐stained sections was quantified in four sections with three different defects per treatment. H&E stains were examined and photographed with a Nikon Eclipse E600 microscope and Spot Advanced software (Diagnostic Instruments, Sterling Heights, MI, USA). Then, six images of three slices of each specimen were randomly collected and were randomly collected under the same exposure value at high magnification (×200). And the inner field of the defect area of each H&E staining slice was determined under low magnification (×40). The area (mm^2^) percentages of the new bone to the area of the defect were calculated using Image‐Pro Plus bioimage professional analysis software. Furthermore, the boundary of the defects around the tooth root was defined, and the new alveolar bone, PDL and cementum were observed.

### Statistical analysis

Statistically significant differences (*P* < 0.05) between the various groups were evaluated by analysis of variance. All statistical analyses were carried out using the spss 19.0 statistical software package (SAS, Cary, NC, USA). All data are expressed as the mean ± standard deviation.

## Results

### Rat PDLSC culture

**Figure 1:**
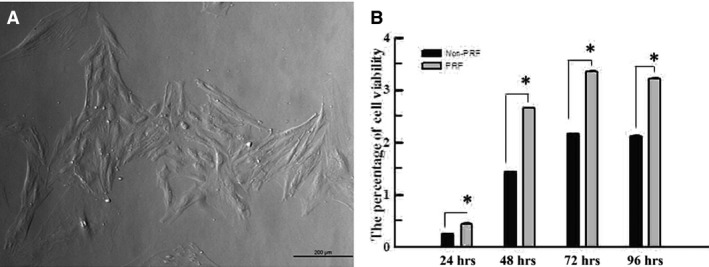
(**A**) The colonies of rat PDLSCs could be observed microscopically, and cells in good condition exhibited a fusiform shape with an oval nucleus, lying in the middle of the cytoplasm (40×). (**B**) MTT assay indicated that the proliferation level of rat PDLSCs in PRF group was higher than that in normal group at 24, 48, 72 and 96 hrs *in vitro*. *Statistically significant difference compared to control and PRF group (*P* < 0.05).

Colonies of rat PDLSCs could be observed microscopically, and cells in good condition exhibited a fusiform shape with an oval nucleus situated in the centre of the cytoplasm (Fig. 1A).

### PRF promotes the proliferation and differentiation of PDLSCs

**Figure 2:**
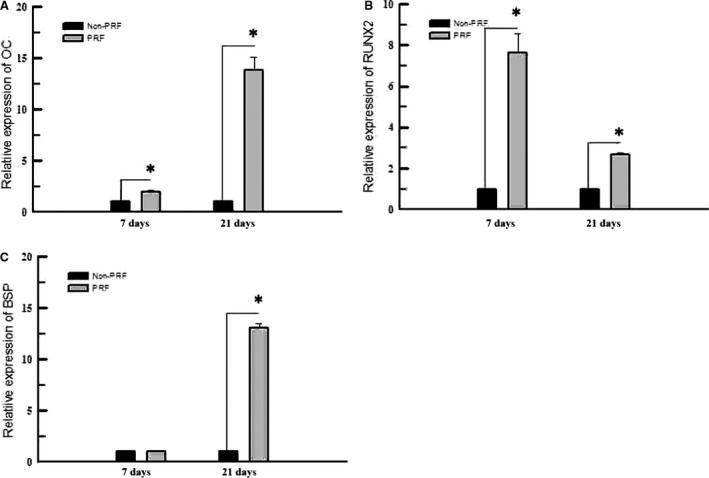
RT‐PCR was used to measure the mRNA expression levels of BSP, OC and *Runx2* of cells cultured in different media after 7 and 14 days. Expressions of OC and *Runx2,* both significantly increased in PRF group, compared with those exposed to the control group at both 7 and 14 days (**A**,** B**). The mRNA expression level of BSP was significantly increased in the cells cultured in the PRF medium at 14 days (**C**). *Statistically significant difference compared to control and PRF group (*P* < 0.05).

**Figure 3:**
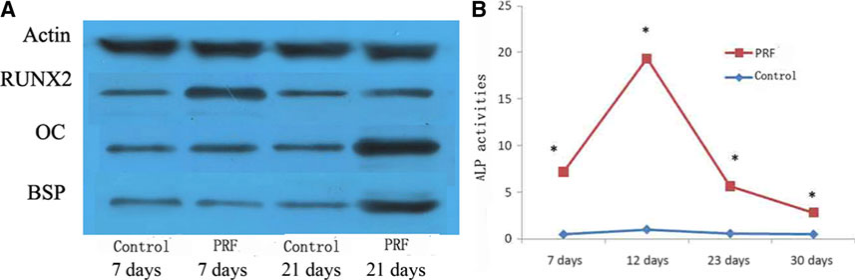
(**A**) Western blot analysis was used to measure the protein expression levels of BSP, OC and *Runx2* of the cells cultured in different media after 7 and 21 days. The results showed that the protein expression levels of OC and *Runx2* were greatly increased in the cells grown in the PRF media at 7 days and those of BSP, OC and *Runx2* were all increased at 21 days. (**B**) ALP activity measurement showed higher ALP activities of rat PDLSCs in the PRF group than those in the control group at 7, 12, 23 and 30 days. *Statistically significant difference compared to control and PRF group (*P* < 0.05).

The MTT assay indicated that PRF promotes the proliferation of rat PDLSCs *in vitro* (Fig. 1B). RT‐PCR was used to measure the mRNA expression levels of BSP, OC and RUNX2 in cells cultured in different media after 7 and 14 days. The mRNA expression of OC and RUNX2 markedly increased in cells exposed to PRF media compared with that in those exposed to control medium at both 7 and 14 days (Fig. 2A and B). In addition, the mRNA expression of BSP significantly increased in cells cultured in PRF media at 14 days (Fig. 2C). Western blot analysis was used to measure BSP, OC and RUNX2 protein levels in cells cultured in different media after 7 and 21 days. OC and RUNX2 protein levels greatly increased in the cells grown in the PRF media at 7 days and those of OC, RUNX2 and BSP increased at 21 days (Fig. 3A, Table 1). In addition, ALP activity increased in cells treated with PRF at 7, 12, 23 and 30 days (Fig. 3B).

**Table 1:** Greyscale value of protein electrophoresis band of BSP, OC and *Runx2* of the cells cultured in different media after 7 and 21 days. (group means ± S.D.; *n* = 3)

	7 days		21 days	
	Control	PRF	Non‐PRF	PRF
Actin	95 ± 2	96 ± 2	94 ± 1	96 ± 1
*Runx2*	56 ± 1	81 ± 2^a^	56 ± 1	55 ± 1
OC	58 ± 2	68 ± 1^a^	67 ± 1	95 ± 2^a^
BSP	50 ± 1	41 ± 2	44 ± 1	87 ± 1^a^

a Statistically significant difference compared to control and PRF group (*P *<* *0.05).

**Figure 4:**
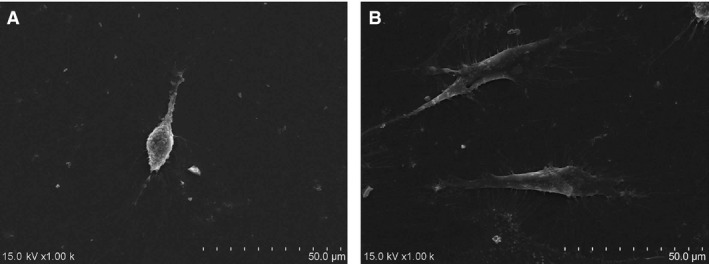
(**A**) Scanning electron microscopy showed that cells cultured in the control medium had a long and thin spindle shape, with tiny, short and less bumps stretching out of the cellular surface. (**B**) By contrast, cells cultured in the PRF medium were larger and showed a fusiform shape, with radial, longer and many more bumps stretching out of the cellular surface.

Scanning electron microscopy showed that cells cultured in control medium had a long, thin spindle shape, with decreased numbers of short, tiny bumps stretching out from the cellular surface (Fig. 4A). By contrast, cells cultured in PRF medium were larger and showed a fusiform shape, with increased numbers of longer radial projections stretching out from the cellular surface (Fig. 4B).

### Increased expression of BSP, OC and RUNX2 mRNA after treatment with PRF combined with PDLSCs

**Figure 5:**
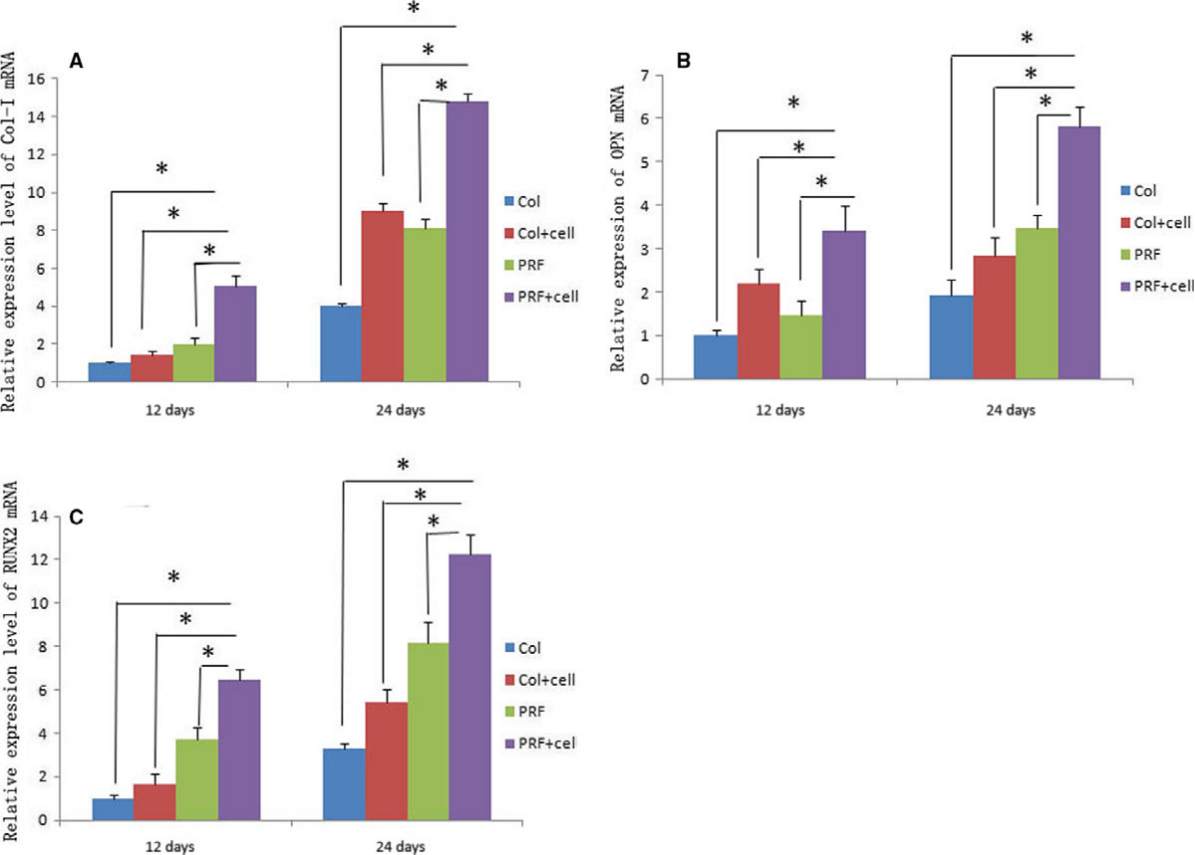
RT‐PCR was performed to measure the mRNA expression levels of COL1A, Opn and RUNX2 in different groups. All bone markers showed higher expression levels at 24 days than at 12 days. In addition, the PRF + cells group showed the highest mRNA expression levels of Col1a, Opn and RUNX2 at both 12 and 24 days (**A**,** B**,** C**). *Statistically significant difference (*P* < 0.05).

To examine the effect of PRF transplantation during new bone formation, total RNA was isolated from all experimental groups. RT‐PCR was performed to measure the mRNA expression levels of COL1A, Opn and RUNX2. All three bone markers showed higher expression at 24 days than at 12 days, and the PRF + cells group showed the highest mRNA expression of COL1A, Opn and RUNX2 at both 12 and 24 days (Fig. 5).

### PRF combined with PDLSCs enhances new bone regeneration

**Figure 6:**
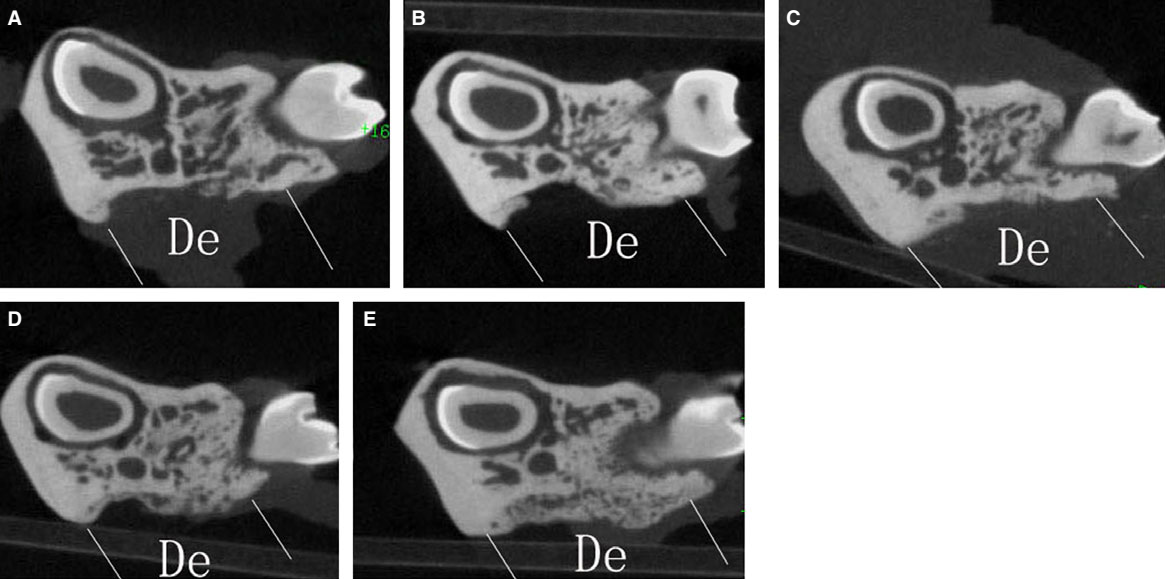
Micro‐CT analysis demonstrated that the sample of the PRF + cells group showed much more newly formed hard tissue than those of the other groups at a similar layer and that of the blank group showed the least amount of new bone formation.

**Figure 7:**
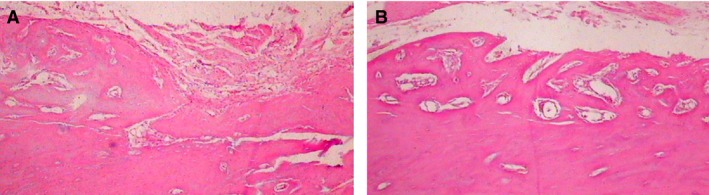
(**A**) At 24 days after surgery, the defect sites in the collagen + cells group were filled with connective tissue and blocked new bone formation at the defect area (100×). (**B**) In the PRF + cells group, the defect sites exhibited marked bone formation, which almost completely replaced the whole bone defects with new bone spicules (100×).

**Figure 8:**
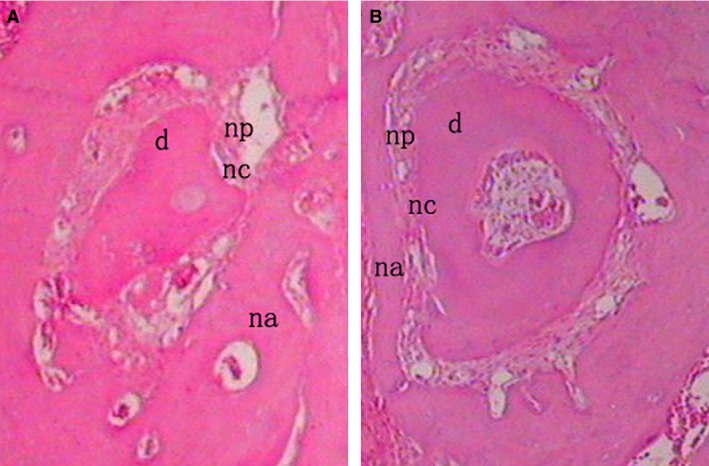
In the PRF group (**A**), little new cementum could be observed at the margin of the defect and the PRF + cells group (**B**) showed a thin layer of new cellular cementum covering the root‐denuded surface at 24 days after surgery. All of the new cementum was cellular cementum, and cracks between the new cementum and the root dentin were observed in some cases. The regenerated PDL fibres separating the new bone from the new cementum was disordered and not perpendicular to the root surface in the PRF + cells group (**B**) and those in the PRF group (**A**) were sparse and loose without periodontal fibre bundle formation at 24 days after surgery (×200). d, dentin; np, new periodontal ligament; na, new alveolar bone; nc, new cementum.

Micro‐CT analysis demonstrated that samples from the PRF + cells group showed much more newly formed hard tissue than the other groups at similar layers. Not surprisingly, the blank group showed the least amount of new bone formation (Fig. 6). Histomorphometric analysis demonstrated that the new bone area percentage in the PRF + cells group was higher than that of other groups 12 and 24 days after surgery (Table 2). At 24 days after surgery, defect sites in the collagen + cells group were filled with connective tissue, which blocked new bone formation (Fig. 7A). By contrast, in the PRF + cells group, the defect sites exhibited marked bone formation, which almost completely replaced the bone defects with new bone spicules (Fig. 7B). Histological analysis demonstrated that the new bone area percentages in the PRF + cells group were significantly greater than those in other groups (*P* < 0.01). The PRF + cells group (Fig. 8B) showed that a thin layer of new cellular cementum (5–10 μm) covering the root‐denuded surface and the regenerated PDL fibres separating the new alveolar bone from the new cementum was disordered and not perpendicular to the root surface. Cracks between the new cementum and the root dentin were observed at high magnification in some cases. In the PRF group (Fig. 8A), little new cementum could be observed at the margin of the defect and new periodontal ligament fibres were sparse and loose without periodontal fibre bundle formation at 24 days after surgery.

**Table 2:** New bone area percentage (group means ± S.D.; *n* = 6)

	12 days	24 days
Collagen	5.54 ± 0.54	23.66 ± 1.48
PRF	7.48 ± 0.99	34.19 ± 2.01
Collagen + cells	8.63 ± 1.17	39.74 ± 2.12
PRF + cells	16.15 ± 2.52^a^^,^ b ^,^ c	62.81 ± 2.63^a^^,^ b ^,^ c

a Statistically significant difference compared to Collagen + cells and PRF + cells group (*P *<* *0.05).

b Statistically significant difference compared to PRF and PRF + cells group (*P *<* *0.05).

c Statistically significant difference compared to Collagen and PRF + cells group (*P *<* *0.05).

## Discussion

PRF is present in platelets in gel form and is rich in various growth factors, including transforming growth factor‐beta 1 (TGFβ‐1), platelet‐derived growth factor (PDGF), insulin‐like growth factor (IGF), vascular endothelial growth factor (VEGF), fibroblast growth factor, epidermal growth factor (EGF) and hepatocyte growth factor. It promotes the movement, proliferation and differentiation of stem cells, as well as neovascularization and collagen synthesis. In addition, PRF can promote bone tissue and soft tissue growth to accelerate wound healing and improve healing quality. TGFβ‐1 triggers fibrosis 26, PDGF contributes to the migration and survival of mesenchymal cells 27, IGF prevents cells from undergoing apoptosis 28, VEGF stimulates vasculogenesis and angiogenesis 29 and EGF functions in cell proliferation and differentiation 30.

Many experimental studies have demonstrated that PRF stimulates the growth of multiple mesenchymal cell types, including fibroblasts 31, dermal prekeratinocytes, preadipocytes, osteoblasts 32, dental pulp cells 33 and bone marrow‐derived mesenchymal stem cells (BMSCs) 34. Studies have also shown that PRF can increase the expression of protein kinase and osteoprotegerin in osteoblasts and could promote osteoblast proliferation, indicating the potential to promote new bone formation 35. Previous studies have also suggested that PRF can induce stem cells to differentiate into cells with osteoblastic (*e.g*. BMSCs) and osteo/odontoblastic (*e.g*. dental pulp cells) phenotypes *in vitro*. In this study, PRF significantly promoted the proliferation of rat PDLSCs and also greatly enhanced their ALP activity and OC, RUNX2 and BSP mRNA and protein levels, thus promoting the osteogenetic differentiation and mineralization of cells.

In our *in vivo* experiments, RT‐PCR showed that the mRNA expression of COL1A, RUNX2 and *Opn* increased from 12 to 24 days. The PRF + cells group showed the highest expression of COL1A, *Opn* and RUNX2, and mRNA levels in PRF groups were higher than those in the collagen groups at both 12 and 24 days. Micro‐CT and histological analysis showed that the PRF + cells group had a significantly greater amount of new alveolar and mandibular bone 24 days after transplantation of rat PDLSCs combined with PRF membrane. These results suggest that PRF may be a better choice than collagen as an autologous biological material in tissue engineering for periodontal regeneration. In addition, there was more active bone formation activity in the PRF + cells group. A slow but substantial release of growth factors from PRF may have played important roles in increasing the cell numbers and inducing their differentiation. Local factors or surrounding primary bone‐derived cells may also play a role in this process. PRF can not only utilize its mechanical effects to postpone the formation of platelets and a variety of cytokines in the blood circulation, but also chemically associates with small molecules, such as various cytokines in the circulation through the glucose glycosaminoglycans, resulting in the slow and substantial release of cytokines during the PRF degradation process of PRF, thereby prolonging the action time of these factors 6, 8. And the fibrin in PRF provides a site for the proliferation and differentiation of tissue‐related cells and plays an important role as a scaffold during tissue repair. PRF is a translucent membrane‐like structure, and a large number of white blood cells are distributed in the fibrin network. These leucocytes continuously release immunoregulatory‐related cytokines during the degradation process, which reduces local inflammation and enhances the host's ability to resist infection. To minimize species and individual differences and imitate clinical transplantation to maximum extent, our *in vivo* experiment implanted PRF and PDLSCs, both derived from rats, into defects in rats.

The ultimate goal of periodontal regeneration is to establish a new periodontal attachment, which means the formation of new cementum with inserting collagen fibres of previously periodontitis involved root surfaces possibly with a concomitant regeneration of supporting alveolar bone. The formation of new cementum is important for new periodontal ligament attachment. It is possible that cementum components have the potential to participate in the regulation of homeostasis and regeneration of the gingiva, PDL and alveolar bone tissues 36. Cracks were observed between some of the new cementum and the root dentin at high magnification. Previous studies have suggested that these cracks may be caused by changes in the position of the specimen during the process of tissue demineralization 37. The presence of sections between the cracks indirectly verified this point. However, this observation also demonstrated that the connections between the new cementum and the original root dentin were rather loose. In our study, at 24 days after surgery, PRF + cells groups showed a thin layer of cellular cementum and in PRF group, only little new cementum could be observed at the margin of the defect. This indicated that cells which contributed to the formation of cementum may derive from the cementoblasts lying in cellular cementum around the defect or PDLSCs implanted. Compared to the PRF group, there was much more new PDL formation. However, there were few new PDL fibre bundles perpendicular to the root surface, and which meant a loose connection between the new PDL and the new cementum or the new alveolar bone. Thus, the quality of the new attachment was poor or in a sense a real periodontal attachment was not established.

In a conclusion, PRF combined with rat PDLSCs can greatly enhance the restoration of rat periodontal defects, by promoting the formation of cementum, alveolar bone and PDL fibres. Further research should be considered to improve obtaining new normal PDL fibres in periodontal regeneration. Animal model of chronic periodontitis also should be considered in the future research, in which the periodontal condition would be more complicated.

## Conflict of interests

All authors declare that we do not have any commercial or associative interest that represents a conflict of interest in connection with the work submitted.
